# Bioaccumulative and conchological assessment of heavy metal transfer in a soil-plant-snail food chain

**DOI:** 10.1186/1752-153X-6-55

**Published:** 2012-06-15

**Authors:** Dragos V Nica, Marian Bura, Iosif Gergen, Monica Harmanescu, Despina-Maria Bordean

**Affiliations:** 1 Banat's University of Agricultural Sciences and Veterinary Medicine from Timisoara, Faculty of Animal Sciences and Biotechnologies, Timisoara, Calea Aradului 119, RO, 300645, Romania; 2 Banat's University of Agricultural Sciences and Veterinary Medicine from Timisoara, Faculty of Food Processing Technology, Calea Aradului 119, RO 300645, Timisoara, Romania; 3 Banat's University of Agricultural Sciences and Veterinary Medicine from Timisoara, Faculty of Agriculture, Timisoara, RO, 300645, Calea Aradului 119, Romania

**Keywords:** Helix pomatia, Bioindicator, Environmental monitoring, Heavy metal accumulation, Food chain, Risk assessment

## Abstract

**Background:**

Copper (Cu), zinc (Zn), cadmium (Cd), and lead (Pb) can pose serious threats to environmental health because they tend to bioaccumulate in terrestrial ecosystems. We investigated under field conditions the transfer of these heavy metals in a soil-plant-snail food chain in Banat area, Romania. The main goal of this paper was to assess the Roman snail (*Helix pomatia*) usefulness in environmental monitoring as bioindicator of heavy metal accumulation. Eight sampling sites, selected by different history of heavy metal (HM) exposure, were chosen to be sampled for soil, nettle leaves, and newly matured snails. This study also aimed to identify the putative effects of HM accumulation in the environment on phenotypic variability in selected shell features, which included shell height (SH), relative shell height (RSH), and whorl number (WN).

**Results:**

Significantly higher amounts of HMs were accumulated in snail hepatopancreas and not in foot. Cu, Zn, and Cd have biomagnified in the snail body, particularly in the hepatopancreas. In contrast, Pb decreased when going up into the food chain. Zn, Cd, and Pb correlated highly with each other at all levels of the investigated food chain. Zn and Pb exhibited an effective soil–plant transfer, whereas in the snail body only foot Cu concentration was correlated with that in soil. There were significant differences among sampling sites for WN, SH, and RSH when compared with reference snails. WN was strongly correlated with Cd and Pb concentrations in nettle leaves but not with Cu and Zn. SH was independent of HM concentrations in soil, snail hepatopancreas, and foot. However, SH correlated negatively with nettle leaves concentrations for each HM except Cu. In contrast, RSH correlated significantly only with Pb concentration in hepatopancreas.

**Conclusions:**

The snail hepatopancreas accumulates high amounts of HMs, and therefore, this organ can function as a reliable biomarker for tracking HM bioavailability in soil. Long-term exposure to HMs via contaminated food might influence the variability of shell traits in snail populations. Therefore, our results highlight the Roman snail (*Helix pomatia*) potential to be used in environmental monitoring studies as bioindicator of HM pollution.

## Background

The growing awareness and concerns about the impact of global pollution on human lives and health have triggered scientists interest to characterize and monitor the quality of the biota. Environmental monitoring is defined as “a time series of measurements of physical, chemical, and/or biological variables, designed to answer questions about environmental change” [1]. Chemical monitoring relies on specific analytical techniques (e.g., titrimetric methods, atomic spectroscopy, mass spectrometry, chromatography) to measure the precise level of specific pollutants in natural environments, but it cannot account for the cumulative impact of complex mixtures [2,3]. Biological monitoring uses instead selected biological responses (e.g., morphological alterations, cellular biomarkers, behavioral changes, etc.) deriving from environmental exposure to complex mixtures of chemicals for evaluating exposure and health risk compared to an appropriate reference [4]. These two complementary approaches have been successfully integrated in most environmental monitoring programs to provide information feedback on the actual impact of human-induced pollution on the environment, and to reflect the efficiency of prescribed mitigation measures to protect the environment [5].

Besides the persistent organic pollutants, heavy metal (HM) accumulation in air, soil, water, and biota is increasingly becoming a global problem with the development of industry, mining activity, application of sewage sludge, and irrigation of waste water [2]. HM pollution of the soil is often less visible and direct than other types of land pollution (e.g., garbage, refuse, littering), but its effects on terrestrial ecosystems and humans are long-lasting and severe [5]. HM toxicity in the environment depends on several physicochemical and biological factors, among which HM bioavailability in soil plays a crucial role in their accumulation along the food chain; therefore, this factor becomes an important issue in assessing the environmental quality [6]. It was also found that synergic and antagonic relationships existing between HM metal ions may increase their toxic potential [7,8]. Such findings are extremely important, because in the environment HMs usually co-occur in complex mixtures [9].

Copper (Cu), zinc (Zn), cadmium (Cd), and lead (Pb) were chosen because they are among the most common HMs which can pollute the environment, especially in areas with high anthropogenic pressure. People have been exposed to these HMs on large scale over the last century, and all of them are known to pose serious threats to terrestrial ecosystems [5]. The Romanian Soil Quality regulations set up the alert threshold level (ATV) to which these HMs accumulate in soil: Cu: 100 mg kg^-1^ d.w.; Zn: 300 mg kg^-1^ d.w.; Cd: 3 mg kg^-1^ d.w.; Pb: 50 mg kg^-1^ d.w. – the values are expressed as miligram per kilogram dry weight (mg kg^-1^ d.w.) [10]. Cu and Zn have essential physiological and biochemical functions in plants and animals [11,12], but excessive levels can be damaging to environmental health [3]. For example, Cu(II) ions (> 40 mg kg^-1^ d.w.) significanly inhibit the alfalafa (*Medicago sativa* L.) seed capacity to germinate and grow [13] whereas exposure to high Zn levels in soil (> 500 mg kg^-1^ d.w.) reduce the ability of plants to absorb iron (Fe) and manganese (Mn) [14]. In contrast, Cd and Pb have no known vital or beneficial effect on organisms [15], except for diatoms where a Cd-based enzyme plays an essential role in regulating atmospheric carbon [16]. Pb and Cd are toxic, persistent, and non-biodegradable in the environment, and hence, they can be easy bioaccumulated and biomagnified along terrestrial food chains [5]. Because all these HMs pose a serious toxic threat to human health and ecosystem integrity, the EU Council Directive 76/464/EEC of 4 May 1976 included these HMs among the most dangerous substances discharged into the natural environment.

The Banat region is a highly industrialized area in western Romania, which includes the studied areas. The diverse mineral resources of this region have facilitated the extensive development of mining, mineral, and metalurgical industry, particularly in the county of Caras-Severin. Most of these industrial facilities were established under Habsburg administration in the eighteenth century and continued till the end of the tweentieth century (e.g., Resita, Otelu Rosu, Anina, Bocsa, etc.) [17]. Long-term pollution in the county of Caras-Severin resulted in Cu, Zn, Cd, and Pb accumulation in soil and vegetation [18]. Another major industrial area in Banat region is the town of Timisoara, the largest and the most populated city from this area. Recent studies occasionally revealed significant Cu, Zn, Cd, and Pb levels in air, soil, water, and vegetation in Timisoara and the neighbouring areas [19]. However, little information is available concerning the bioaccumulation and the biomagnification of these HMs in terrestrial ecosystems in Romania, particularly in Banat region.

Many studies [20-23] showed that land snails can concentrate high levels of Cu, Zn, Cd, and Pb in their soft tissue without revealing any major metabolic disorders. These HMs were found to accumulate into the snail shell, often resulting in alterations of shell geometry or size [20,24-26]. In polluted soils Cu, Zn, Cd, and Pb are taken by vegetation, and subsequently they can be transfered along food chain to herbivorous organisms. In addition, Cu, Zn, Cd, and Pb accumulate in dead plant and animal material, and therefore, they are ingested by organisms that feed on decaying organic matter (i.e., detritivore organisms). Because snails act on trophic level, both as herbivorous and detritivore organisms, they can be involved in these HM biomagnification along the food chain [22,27]. Overall, terrestrial gastropods have the potential to be used in environmental monitoring as model invertebrate for Cu, Zn, Cd, and Pb accumulation in terrestrial ecosystems [28].

The present research investigates land snails as endpoints of Cu, Zn, Cd, and Pb transfer in a soil-plant-herbivore food chain in terrestrial ecosystems of the Banat region. It compares the accumulation of these HMs in eight populations of snails from habitats exposed to industrial pollution for more than 30 years. By recording Cu, Zn, Cd, and Pb level in soil, plant, and snail for each population, HM biomagnification along food chain is traced to assess the potential of native gastropods to be used in environmental monitoring studies. In addition, we have examined the influence of long-term of HM exposure on intraspecific variation in shell morphology(shell size, relative shell height, whorl number).

## Results and discussions

### HM accumulation and transfer in soil–nettle-snail food chain

#### HM accumulation in soil

**Figure 1:**
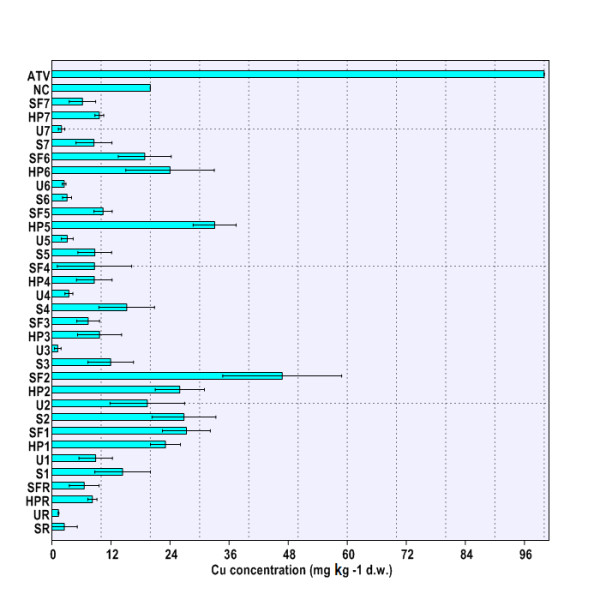
**Cu concentration in environmental matrices (soil, nettle leaves, snail body).** Legend. SR – HM concentrations in soil at reference site (THR), UR – HM concentrations in nettle leaves at reference site (THR), HPR – HM concentrations in snail hepatopancreas at reference site (THR), SFR – HM concentrations in snail foot at reference site (THR), S1-S7 – HM concentrations in soil at sites 1-7 (THM1-THM7), U1-U7 – HM concentrations in nettle leaves at sites 1-7 (THM1-THM7), HP1-HP7 – HM concentrations in snail hepatopancreas at sites 1-7 (THM1-THM7), SF1-SF7 – HM concentrations in snail hepatopancreas at sites 1-7 (THM1-THM7), ATV – alert threshold level (HM concentration in soil). NC – normal content level (HM concentration in soil).

**Figure 2:**
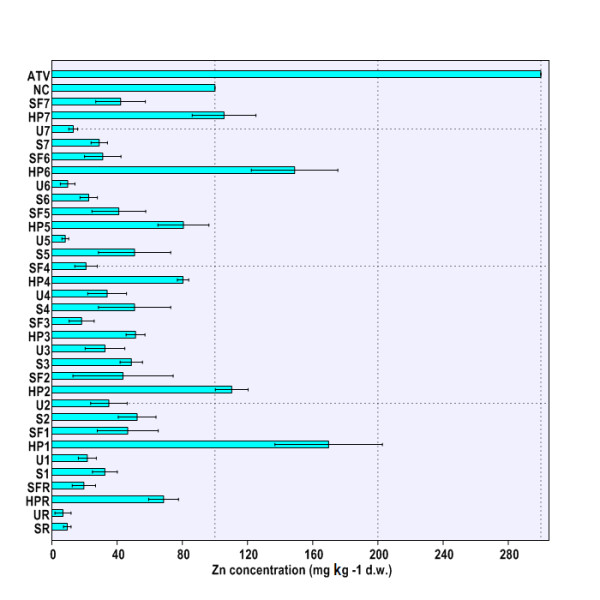
**Zn concentration in environmental matrices (soil, nettle leaves, snail body).** Legend. SR – HM concentrations in soil at reference site (THR), UR – HM concentrations in nettle leaves at reference site (THR), HPR – HM concentrations in snail hepatopancreas at reference site (THR), SFR – HM concentrations in snail foot at reference site (THR), S1-S7 – HM concentrations in soil at sites 1-7 (THM1-THM7), U1-U7 – HM concentrations in nettle leaves at sites 1-7 (THM1-THM7), HP1-HP7 – HM concentrations in snail hepatopancreas at sites 1-7 (THM1-THM7), SF1-SF7 – HM concentrations in snail hepatopancreas at sites 1-7 (THM1-THM7), ATV – alert threshold level (HM concentration in soil). NC – normal content level (HM concentration in soil).

**Figure 3:**
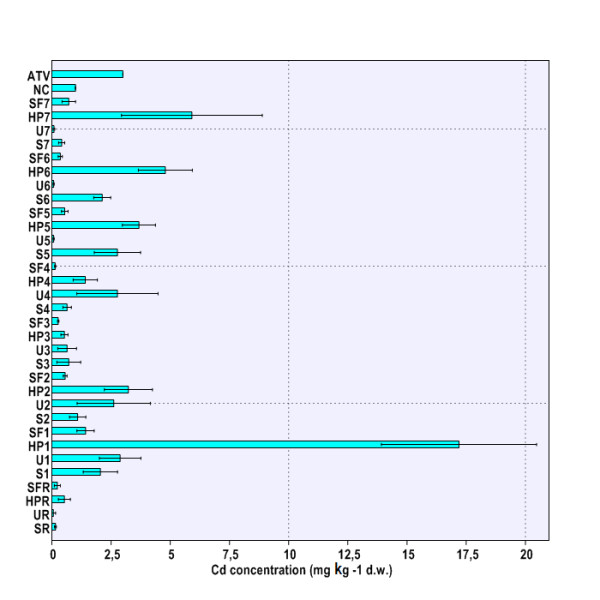
**Cd concentration in environmental matrices (soil, nettle leaves, snail body).** Legend. SR – HM concentrations in soil at reference site (THR), UR – HM concentrations in nettle leaves at reference site (THR), HPR – HM concentrations in snail hepatopancreas at reference site (THR), SFR – HM concentrations in snail foot at reference site (THR), S1-S7 – HM concentrations in soil at sites 1-7 (THM1-THM7), U1-U7 – HM concentrations in nettle leaves at sites 1-7 (THM1-THM7), HP1-HP7 – HM concentrations in snail hepatopancreas at sites 1-7 (THM1-THM7), SF1-SF7 – HM concentrations in snail hepatopancreas at sites 1-7 (THM1-THM7), ATV – alert threshold level (HM concentration in soil). NC – normal content level (HM concentration in soil).

**Figure 4:**
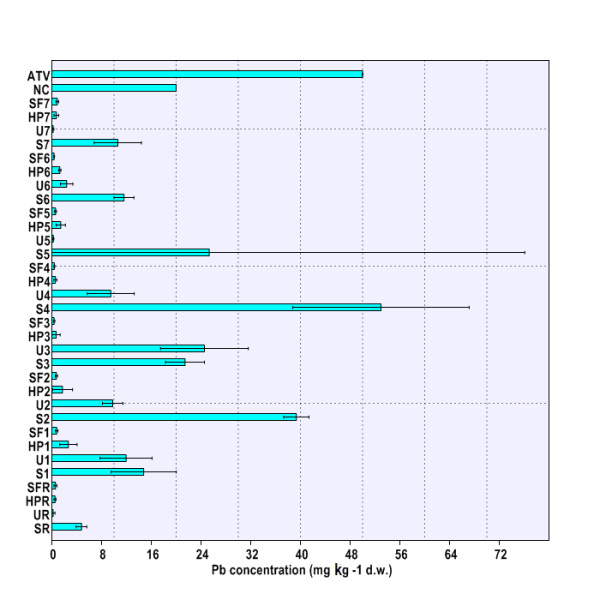
**Pb concentration in environmental matrices (soil, nettle leaves, snail body).** Legend. SR – HM concentrations in soil at reference site (THR), UR – HM concentrations in nettle leaves at reference site (THR), HPR – HM concentrations in snail hepatopancreas at reference site (THR), SFR – HM concentrations in snail foot at reference site (THR), S1-S7 – HM concentrations in soil at sites 1-7 (THM1-THM7), U1-U7 – HM concentrations in nettle leaves at sites 1-7 (THM1-THM7), HP1-HP7 – HM concentrations in snail hepatopancreas at sites 1-7 (THM1-THM7), SF1-SF7 – HM concentrations in snail hepatopancreas at sites 1-7 (THM1-THM7), ATV – alert threshold level (HM concentration in soil). NC – normal content level (HM concentration in soil).

Our results showed that Cu, Zn, Cd, and Pb concentrations in soil were significantly different among sampling sites (Cu, Zn, Cd: p < 0.01; Pb: p < 0.05). The lowest HM values were registered for the site THR (as shown in Figure 1, 2, 3, 4), and therefore, they validated the choosing of this location as the reference site. The maximal Cu and Zn loadings in soil were found at site THM2 (Figure 1, 2). Soil Cd concentration exhibited the highest value at site THM5 (Figure 3), whereas the maximal amount of Pb in soil was detected at site THM4 (Figure 4). Soil HM concentrations were comparable with results of previous pedological studies in Banat area [18,19,29,30]. However, measured values were generally lower than in other areas exposed to intense anthropogenic pressure and pollution. Therefore, similar studies that investigated HM food chain transfer to land snails in Copsa Mica region, Romania [31] and the Biesboch flood-plain area, the Netherlands [22] frequently found 5-10 times higher levels of HM accumulation in background soils. These differences were explained by the different level and history of HM exposure. Both Copsa Mica and Biesboch areas are well known in Europe as ‘hotspots’ of HM pollution [32,33] whereas the Banat area may be regarded as a low to moderate HM polluted area [34].

HM concentrations within the soil compartment, as shown in Table 1, correlated highly with each other, except for Cu (r = 0.81-0.94, 0.05 < p < 0.001). Similar metal-metal interactions in soil (r = 0.91-0.95, p < 0.001) have also been reported in the Biesboch area between Cu, Zn, Cd, and Pb [22]. The normal content (NC) and the alert threshold level (ATV) for each investigated HM were used to assess the risks posed by soil HM contamination on environmental health. The values were compiled from the Romanian Soil Quality regulations [10]. It was found that soil Cu and Zn concentrations were generally within NC values at all sites (Figure 1, 2). Although in several cases the soil Cd concentrations at sampling sites did exceed NC levels (THM1, THM5, THM6), the measured values did not reach ATV level (Figure 3). It was found that only Pb content slightly did exceed ATV value at site THM4 (Figure 4). Hence, in accordance with Romanian Soil Quality regulations [10] it was concluded that all snails originated in habitats with low HM accumulation in soil. However, heightened Cd levels at sites THM1, THM5 and THM6 (Figure 3) are expected to raise serious environmental concerns if their level in the near future reaches ATV value.

**Table 1:** Correlations between Cu, Zn, Cd, and Pb concentrations in soil, nettle leaves, and snail body

	**Log**	**Log**	**Log**	**Log**	**Log**	**Log**	**Log**	**Log**	**Log**	**Log**	**Log**	**Log**	**Log**	**Log**	**Log**	**Log**
	**Cu(S)**	**Zn(S)**	**Cd(S)**	**Pb(S)**	**Cu(U)**	**Zn(U)**	**Cd(U)**	**Pb(U)**	**Cu(SF)**	**Zn(SF)**	**Cd(SF)**	**Pb(SF)**	**Cu(HP)**	**Zn(HP)**	**Cd(HP)**	**Pb(HP)**
**Log Cu(S)**		0.95***	0.31 ns	0.81*	0.67 ns	0.85**	0.83*	0.61 ns	0.45 ns	0.34 ns	0.34 ns	0.26 ns	0.20 ns	0.05 ns	0.25 ns	0.37 ns
**Log Zn(S)**			0.22 ns	0.88**	0.45 ns	0.93**	0.80*	0.69 ns	0.25 ns	0.11 ns	0.11 ns	0.03 ns	-0.02 ns	-0.11 ns	0.08 ns	0.15 ns
**Log Cd(S)**				0.25 ns	0.47 ns	0.11 ns	0.18 ns	0.31 ns	0.59 ns	0.58 ns	0.58 ns	-0.07 ns	0.87**	0.52 ns	0.64 ns	0.81*
**Log Pb(S)**					0.52 ns	0.90**	0.82*	0.76*	0.38 ns	-0.02 ns	-0.02 ns	-0.23 ns	0.029 ns	-0.01 ns	0.02 ns	0.10 ns
**Log Cu(U)**						0.46 ns	0.66 ns	0.40 ns	0.90**	0.67 ns	0.50 ns	0.46 ns	0.62 ns	0.60 ns	0.58 ns	0.75*
**Log Zn(U)**							0.90**	0.87**	0.35 ns	-0.07 ns	-0.07 ns	-0.13 ns	-0.12 ns	-0.07 ns	-0.03 ns	0.10 ns
**Log Cd(U)**								0.84*	0.52 ns	0.06 ns	0.08 ns	0.04 ns	0.03 ns	0.14 ns	0.14 ns	0.31 ns
**Log Pb(U)**									0.48 ns	-0.16 ns	-0.06 ns	-0.38 ns	0.05 ns	0.07 ns	-0.01 ns	0.27 ns
**Log Cu(SF)**										0.59 ns	0.49 ns	0.24 ns	0.74*	0.67 ns	0.55 ns	0.84**
**Log Zn(SF)**											0.88**	0.74*	0.73*	0.74*	0.90**	0.80*
**Log Cd(SF)**												0.75*	0.59 ns	0.66 ns	0.83*	0.80*
**Log Pb(SF)**													0.21 ns	0.41 ns	0.56 ns	0.39 ns
**Log Cu(HP)**														0.57 ns	0.63 ns	0.89**
**Log Zn(HP)**															0.90**	0.74*
**Log Cd(HP)**																0.80*
**Log Pb(HP)**																

Note.*** – p < 0.001, ** – p < 0.01, * – p < 0.05, ns – p > 0.05.

***Abbreviations.*** Cu(S) – copper concentrations in soil, Cu(U) – copper concentrations in nettle leaves, Cu(SF) – copper concentrations in snail foot, Cu(HP) – copper concentrations in snail hepatopancreas, Zn(S) – zinc concentrations in soil, Zn(U) – zinc concentrations in nettle leaves, Zn(SF) – zinc concentrations in snail foot, Zn(HP) – zinc concentrations in snail hepatopancreas, Cd(S) – cadmium concentrations in soil, Cd(U) – cadmium concentrations in nettle leaves, Cd(SF) – cadmium concentrations in snail foot, Cd(HP) – cadmium concentrations in snail hepatopancreas, Pb(S) – lead concentrations in soil, Pb(U) – lead concentrations in nettle leaves, Pb(SF) – lead concentrations in snail foot, Pb(HP) – lead concentrations in snail hepatopancreas.

#### HM accumulation in nettle leaves

Our investigations revealed significant differences in HM accumulation in nettle leaves at sampling sites (Cu, Zn, Cd, Pb: p < 0.01). The highest Cu concentration in *Urtica dioica* leaves was detected at site THM2 (Figure 1). Elevated levels of Zn were found in nettle leaves at sites THM2, THM3, and THM4 (Figure 2), whereas Cd reached the maximal values at sites THM1, THM2, and THM4 (Figure 3). However, the nettle leaves sampled at site THM3 accumulated, by far, the highest Pb amount (Figure 4). Interestingly, HM loadings in nettle leaves compared fairly well with most reported values in Biesboch area [22], but they were much lower than in Copsa Mica area, particularly for Cd, Zn, and Pb [31]. Therefore, pollution intensity can be an important fostering factor in HM transfer in terrestrial ecosystems.

Our results (Table 1) showed that HM interactions within the leaf compartment followed the same pattern as that found in soil, i.e., strong correlations among Zn, Cd, and Pb level (r = 0.84-0.90, 0.05 < p ≤ 0.001). In contrast, other authors [22] have found that only correlations including Cd were significant at the same level (r = 0.33-0.43, 0.05 < p < 0.001). As shown in Table 1, we identified an effective soil-plant transfer of Zn and Pb in the selected food chain (r = 0.76-0.93, 0.05 < p ≤ 0.001). In Biesboch area, however, only Zn transfer was reported from the soil to the leaf compartment (Zn: r = 0.45, p < 0.001; Cu, Cd, Pb: p > 0.05). Not only the pollution level itself can be held accountable for such diverse and complex data, but also HM bioavailability in soil is essential for HM soil-plant transfer. The dynamics of this process is mainly regulated by soil physico-chemical properties (e.g., total metal content, humidity, clay and hydrous oxide content, organic matter, pH, redox conditions) [35]. Overall, it was suggested that the pattern of HM accumulation and transfer in nettle leaves resulted from the cumulative action of low soil Cd bioavailability to plants [9,19] and significant HM content of soils in Banat area [36].

#### HM accumulation in snail body

The location had a significant influence on HM burden in the snail hepatopancreas (Cu, Zn, Cd, Pb: p < 0.01) and foot (Cu, Zn, Cd, Pb: p < 0.01). Moreover, highly significant differences were found when comparing accumulation of each HM in snail hepatopancreas with that in foot (Cu, Zn, Cd, Pb: p < 0.001). Therefore, one can conclude that HM accumulation in the snail body depends on both the area of origin and investigated body parts (Figure 1, 2, 3, 4). Globally speaking, the snail hepatopancreas concentrated the highest HM amounts at sites THM1, THM2, THM5, and THM6, except for Cd whose level was also reported to be elevated at site TM7 (Figure 1, 2, 3, 4). Similar trends of HM accumulation were found in the snail foot for Cu and Zn, whereas Cd and Pb levels were much more homogenous, regardless of locations (Figure 1, 2, 3, 4). Although the measured values were slightly higher in hepatopancreas, the Cu and Pb levels in the snail body usually varied within the same range (Figure 1, 4). In contrast, Zn and Cd burdens in snail hepatopancreas were always at least 2.0 times higher than in the snail foot (Figure 2, 3). HM concentration registered the maximal relative difference (%) between the hepatopancreas and the foot at site TM1, where the Cd level was more than 10 times higher in the snail hepatopancreas (Figure 1, 4).

The present study attested to Roman snail the ability to concentrate trace metals in its tissues, and therefore, this species was included among the so-called ‘bioindicators of accumulation’ [37]. The accumulation pattern over the different tissue parts (i.e., hepatopancreas vs. foot) was consistent with other studies that revealed the prefferential deposition of most HMs in the *Helix pomatia* hepatopancreas [31,38,39]. When compared the results with those of other field studies, the measured values were generally much lower than in areas with elevated levels and/or exposed to intensive long-term HM pollution [31,38].

Our findings showed that, excepting Pb burden in hepatopancreas, Cu concentrations in snail foot and hepatopancreas were indepedent variables (Table 1). Interestingly, like in soil and leaf compartment, Zn, Cd, and Pb concentrations correlated highly with each other in both hepatopancreas and foot (r = 0.74-0.90, 0.05 < p < 0.001). It was therefore assumed that these HMs have been transferred together in the investigated food chains. These results may have resulted from similar geogenic sources (e.g., composition of parent rock, soil physico-chemical properties), anthropogenic causes (e.g., history of pollution), and/or environmental conditions (e.g., climate, habitat, trophic chain). We hypothesized that such patterns of HM accumulation and transfer in the biota may be used as biochemical fingerprint for tracing and characterizing the environmental pollution risks in a specific area.

In contrast to our study, low and moderate correlations were found among Cu, Zn, Cd, and Pb concentrations in *Cepaea nemoralis* sampled from the Biesboch area (r = 0.37-0.64, 0.05 < p < 0.001), except for Cu-Pb interactions (p > 0.05). In this case, HM accumulation was measured for the whole snail body, excluding the shell [22]. HM pairs Cd-Zn and Cd-Pb correlated moderately with each other in *Helix aspersa* hepatopancreas (r = 0.34-0.54, 0.01 < p < 0.001). Besides these two HM pairs, there were also significant correlations between the whole body Pb and Zn concentrations (r = 0.59-0.65, p < 0.001) as well as between Cu-Zn and Pb-Zn pairs for the whole body without hepatopancreas (r = 0.20-0.73, 0.05 < p < 0.001) [21]. In addition to the exposure to HMs via food uptake, land snails regularly eat soil and they are also exposed to HMs via epithelial contact [40]. As a result, future studies should be designed to verify our hypothesis and to elucidate such complex interactions between HMs during their transfer in the biota.

Cu-Cu, Zn-Zn, and Cd-Cd correlations between the snail hepatopancreas and foot were highly significant (r = 0.73–0.84, 0.05 < p < 0.01), whereas no relationship was found for the Pb content (Table 1). Moderate Cu-Cu correlations were found between the snail foot and soil (Table 1), highlighting the importance of this organ in snail metabolism as an important place of Cu storage [23,41].

Studying HM transfer in a soil-nettle-snail food chain in the Biesboch area, moderate correlations (r = 0.41 – 0.64, 0.05 < p < 0.001) were found for all HMs in adult *Cepaea nemoralis* (whole body, excluding shell), except for Cu-Cu interactions between snail and soil (p > 0.05) [22]. *Helix aspersa* was investigated as sentinel organism to exposure to both physiological and xenobiotic HMs (i.e., Cu, Zn: physiological HMs; Cd, Pb: xenobiotic HMs) in a food chain soil-dandelion-snail. There were found moderate correlations between Zn and Cd concentrations in the snail body (whole body, whole body without hepatopancreas, hepatopancreas) and soil (r = 0.52-0.69, p < 0.001). In the case of Pb, highly significant correlations were detected only when soil concentrations were compared to hepatopancreas and whole body Pb content (r = 0.26 – 0.40, p < 0.001). In contrast, no significant relationship (r = 0.02- 0.08, p > 0.05) was found between soil Cu concentrations and either the whole snail body or snail body parts [21]. Such discrepant results were attributed to different selectivity in choice of food exhibited by these species of land snails and by different bioavailability of HMs in investigated food chains.

#### HM biomagnification along food chain soil-nettle-snail

HM accumulation and transfer from soil to nettle have systematically revealed no metal biomagnification at the first trophic level, the primary producers (*Urtica dioica*; Urticaceae) (Figure 1, 2, 3, 4). In contrast, Cu, Zn, and Cd concentrations at the second trophic level, the primary consumers (*Helix pomatia*; Helicidae), have usually exceeded those found at the first trophic level (Figure 1, 2, 3, 4). Interestingly, except for sites THM5, THM7, and THR, the hepatopancreatic and the foot Pb levels were lower than in the nettle leaves (Figure 4).

Because land snails can accumulate higher levels of Cu and Cd than the environmental concentrations, they are generally recognized as “macroconcentrator” species for these HMs. In contrast, terrestrial gastropods are “microconcentrator” species for Zn and Pb [37]. Our results evidenced that Cu, Zn, and Cd have biomagnified along in snail body, particularly in the snail hepatopancreas, whereas Pb tended to decrease when going up into the food chain. This was explained by the fact that soils from Banat area are naturally rich in Zn [36].

Similar findings (i.e., Cu, Zn, and Cd biomagnification and Pb biodepletion in snail body relative to vegetation concentrations) were reported in the Biesboch area for *Cepaea nemoralis*[22] and in the Copsa Mica area for *Helix pomatia*[31]. Both studies investigated HM accumulation in a soil-nettle-snail food chain. Evaluating the use of *H. aspersa* as sentinel for mapping pollution, it was found that mean vegetation (*Taraxacum* sp.) levels of Cu, Zn, Cd, and Pb were systematically lower than in snail body [21]. Similarly, Zn, Cd, and Pb biomagnified in *H. aspersa* body via autochthonous plants (*Graminaceae*) gathered at the vicinity of a highway [27]. Such examples illustrate the importance of identifying the key particularities of each food chain (e.g., soil physico-chemical properties, HMs bioavailability, diet composition, climatic conditions) before employing the Roman snail (*Helix pomatia*) as bioindicator in environmental monitoring studies in this area. Nevertheless, the sensitivity of *Helix pomatia* as bioindicator organism depends on the investigated body parts. Our study showed that the snail hepatopancreas accumulates significant amounts of HMs, pointing to this organ importance as biomarker of HM pollution.

**Figure 5:**
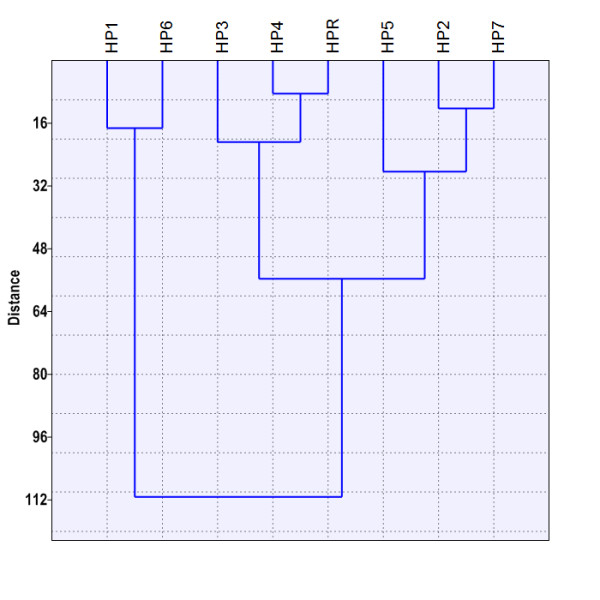
**Dendrogram of the cluster analysis based on HM accumulation in the snail hepatopancreas.** HP1-HP7 – HM concentrations in snail hepatopancreas at sites 1-7 (THM1-THM7); HPR – HM concentrations in snail hepatopancreas at reference site (THR).

The dendrogram (Figure 5) shows the results obtained from using hierarchical cluster analysis and squared Euclidean distance as a criterion of similarity. Therefore, based on HM loading in snail hepatopancreas, the investigated locations can be classified into three main groups (r = 0.81). The first group corresponds to the most polluted areas from the investigated sites (THM1 and THM6). Both areas are well known for long and intensive exposure to HMs. This group is also characterized by the biggest Euclidean distance to the other groups. The second group corresponds to the cleanest areas in terms of HMs accumulated in the snail hepatopancreas. The three sites in this group are either located in low-anthropized areas (THR) or in areas exposed to other types of industrial pollution, particularly to chemical industry (THM3, THM4). The last group includes locations placed near potential sources of air pollution with HMs: THM2 – power plant, THM5 – vehicular traffic, THM7 – steel industry. Therefore, our results suggested that regions with similar HM exposure can be appropriately grouped by applying cluster analysis to HM accumulation in snail hepatopancreas.

### HM influence on shell architecture

#### Estimation of shell size from linear shell dimensions

The internal volume of a snail shell signifies the amount of housing space available for body growth and, thus, this feature is directly associated with the snail size [42]. Two conchological parameters, the shell height (SH) and the shell width (SW), are primarily used to estimate the shell volume (SV) from linear shell dimensions [43,44]; such formulas are species-specific, but little information exists regarding the mathematical relationships for calculating *Helix pomatia* SV.

Measured values for SH and SW were normally distributed (Shapiro-Wilcoxon test, p > 0.05). Although both conchological features correlated significantly with SV, there was a stronger positive relationship between SV and SH (r = 0.80, p < 0.001) than between SV and SW (r = 0.63, p < 0.01). It was therefore concluded that for the investigated snail populations SH is a more accurate predictor of shell size than SW. A similar approach has been already used for assessing shell growth to juvenile *Helix aspersa* exposed to Pb-enriched food [25].

#### Influence of HM accumulation shell traits

Our results confirmed that all conchological features (SH, RSH, WN) were normally distributed (all p`s > 0.05) and satisfied the criterion of homoscedasticity (all p`s > 0.05). The area of origin had a significant influence on shell traits variation among the eight snail populations (p < 0.001). Post hoc analysis revealed that THR snails had significanly larger shells than the snail populations inhabiting locations THM1, THM2, THM3, and THM4. In contrast, no significant differences were found for shell size between THR snails and either THM5 or THM7 snails (Table 2).

**Table 2:** The mean values of conchological features from the eight snail populations

**Sampling site**		**Shell height (SH)**	**Relative shell height (RSH)**	**Whorl number (WN)**
THM1	Timisoara city Landfill	3.63 ± 0.19***	0.98 ± 0.03 ns	4.92 ± 0.04***
	(Parta-Sag/Timis county)			
THM2	South Industrial Platform	3.86 ± 0.11**	0.96 ± 0.02 ns	4.94 ± 0.13***
	(Timisoara/Timis county)			
THM3	North Industrial Platform	3.73 ± 0.24***	1.01 ± 0.04*	4.44 ± 0.15 ns
	(Timisoara/Timis county)			
THM4	East Industrial Platform	3.65 ± 0.20***	0.95 ± 0.02 ns	4.31 ± 0.15*
	(Timisoara/Timis county)			
THM5	Timisoara, Communal Road 64 (DC64)	4.26 ± 0.20 ns	0.96 ± 0.04 ns	4.49 ± 0.14 ns
	(Timisoara/Timis county)			
THM6	National Road Resita-Caransebes (DN58)	3.90 ± 0.17*	0.97 ± 0.03 ns	4.80 ± 0.09***
	(Caras-Severin county)			
THM7	Principal Street	3.99 ± 0.19 ns	0.95 ± 0.04 ns	4.66 ± 0.16**
	(Otelu Rosu/Caras-Severin county)			
THR	Salbagelu Nou	4.18 ± 0.28	0.97 ± 0.03	4.47 ± 0.14
	(Caras-Severin county)			

Note.*** – p < 0.001, ** – p < 0.01, * – p < 0.05, ns – p > 0.05 (Anova, posthoc Dunnett’s test). Figures in bold type defines values significantly lower than the control group (THR), whereas the bold and underlined values are significantly higher than the control group (THR).

It was found that the level of anthropic pollution is often associated with the shell size. Thus, smaller shells were reported for snails inhabiting more polluted habitats [44-46]. *Cepaea nemoralis* displayed a negative correlation (r = -0.61) between the shell volume and the Zn concentration in soil [26]. In our study SH correlated highly with HM concentrations in nettle leaves, except for Cu (Zn: r = - 0.81, p < 0.05; Cd: r = - 0.80, p < 0.05; Pb: r = - 0.89, p < 0.01; Cu: p = 0.687). In contrast, no significant relationships were found between SH and HM concentrations in soil, hepatopancreas, and foot (0.080 < p < 0.892).

When compared with the reference site (THR), RSH was found to be significantly higher only at site THM3, whereas no significant differences were determined for the other snail populations (Table 2). The relative shell height (RSH) of a snail species relates to the angle of the substrate on which activity occurs [47]. Flatter shells facilitate movement on substrates with foliated structures (e.g., leaf litter), whereas higher shells are more advantageous on substrate with a fine particle structure (e.g., soil) [48]. Several studies demonstrated that this feature is primarily genetically determined, but, within a given form, it may also be influenced by phenotype [49]. Therefore, the sampled snails have originated from habitats with different substrates or they belong to different subspecies.

Interestingly, RSH correlated highly with Pb concentrations in the snail hepatopancreas (r = 0.77, p < 0.05), although it systematically revealed no significant relationships with HMs content along the soil-nettle-snail food chain (0.054 < p < 0.487). Long history of Pb-exposure was associated with more robust shells as related to width/height ratio [24]. The aforementioned correlations showed that there are no significant Pb-Pb interactions between snail foot and hepatopancreas. It was therefore suggested that investigated snail populations may have adopted different strategies to deal with exposure to Pb. Such strategies have been already described in the case of *Helix aspersa* for which Pb bioavailability in different populations was reversely related with soft tissue growth rates and food Mg content [50].

The area of origin exhibited a significant bidirectional influence on WN (Table 2). Thus, THM1, THM2, THM6, and THM7 had significantly more whorls than THR snails (p < 0.05), whereas only THM4 snails revealed significantly less whorls (p < 0.05). HM levels in soil, snail hepatopancreas and foot were not correlated with WN (0.054 < p < 0.922), except for soil Cu content which was highly correlated with WN (r = 0.72, p < 0.05). Metabolic needs (i.e., Cu-based respiratory hemocyanins) may explain our results as Cu occurs in the environment at concentrations near the minimum nutritional requirements of invertebrates [51]. According to our results, WN was strongly positively correlated with both Cd and Pb levels in nettle leaves (Cd: r = 0.84, p < 0.01; Pb: r = 0.81, p < 0.01), but displayed no significant relationships with Cu and Zn concentrations in the leaf compartment (Cu: p = 0.058; Zn = 0.059), which has not been reported yet.

When the apertural rim folds back and forms the apertural lip the shell growth ends; thus, from then on WN remains constant throughout the snail life. Calcium carbonate deposition is carried out at a relatively constant rate [52]. In addition, within the same populations snails of similar sizes have equal whorl number [53]. WN can therefore be regarded as a rough estimator of snail maturation age. As a result, it was suggested that long-term exposure to Cd and Pb contaminated food may affect snail populations by decreasing snail size and delaying the maturation age because of increased detoxification effort.

## Conclusions

Given the significant ability to concentrate HMs in its body, the Roman snail (*Helix pomatia*) is a relevant accumulation bioindicator of long-term metallic exposure under field conditions. The snail hepatopancreas proved to be extremely sensitive to HM traces from soil and have frequently accumulated several times higher amounts of Cu, Zn, and Cd than in lower trophic levels (soil and nettle); therefore, this organ can be used for tracking HM bioavailability in soil.

Zn, Cd, and Pb appear to interfere with each other in primary producers and consumers, although this might be attributable to close association in background soils of the eight sites examined here. Therefore, it is suggested that HM accumulation in investigated areas imply a specific biochemical fingerprint. However, HMs in hepatopancreas appear to be independent of concentration in soil and nettle leaves, except for Cu levels in soil and snail foot. In the light of present data, the environmental monitoring with snails must be extended to HM mixtures since such associations are regularly found in soil.

Long-term Cd and Pb uptake via vegetation ingestion is assumed to be associated with smaller shells and higher whorl numbers. Because land snails often exhibit intra-annual cycles of activity interspersed by periods of dormancy, this perspective must be more detailed investigated to establish the real impact of anthopic HM pollution on wild snail populations.

### Experimental

#### Study animals and collecting sites

**Figure 6:**
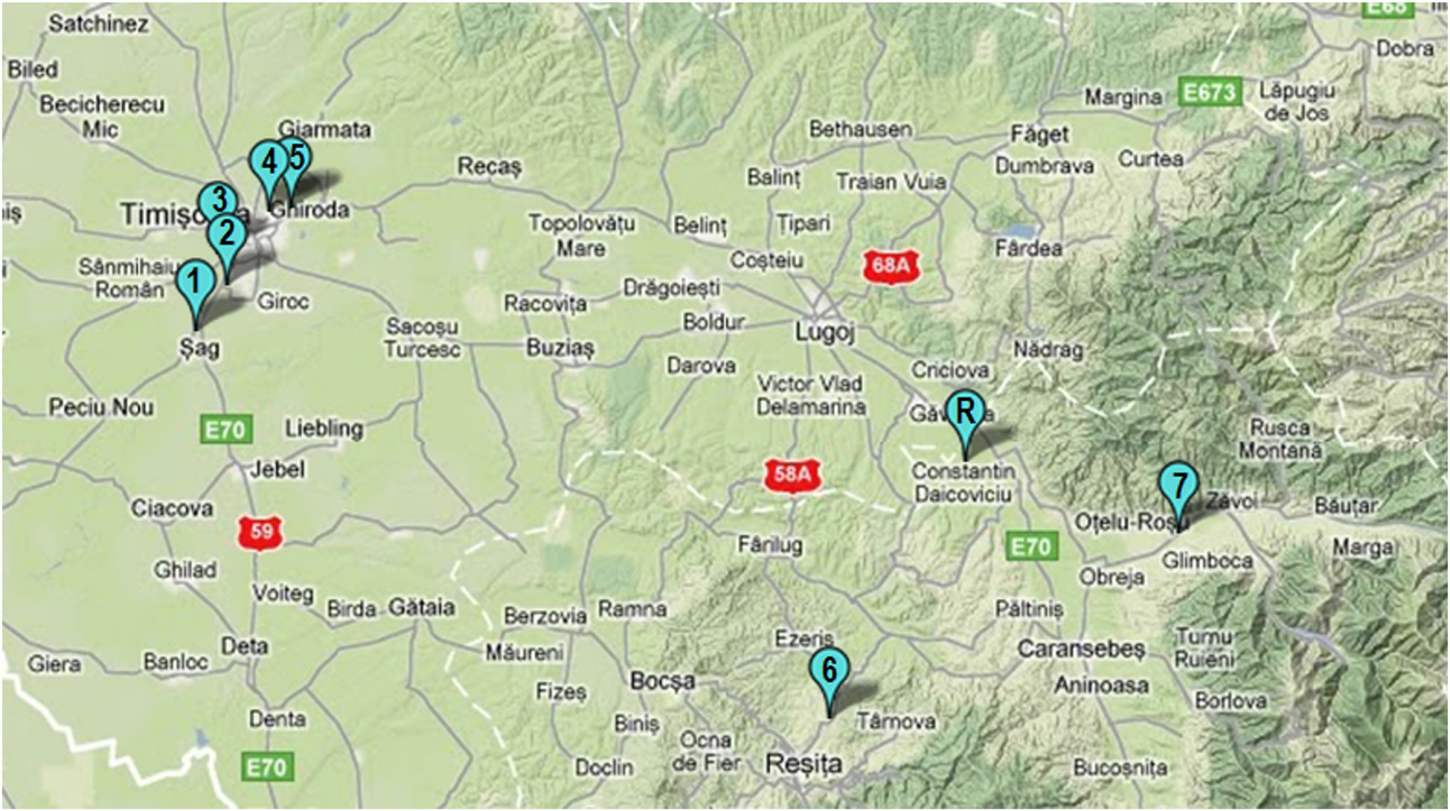
**Map showing the locations of soil, nettle leaves, and snail sampling sites.** Legend. 1 – site THM1; 2 – site THM2; 3 – site THM3; 4 – site THM4; 5 – site THM5; 6 – site THM6; 7 – site THM7; R – site THR.

The Roman snail (*Helix pomatia* Linnaeus, 1758) is a common East and Central European snail species of the family *Helicidae*. In Romania, it inhabits forests, gardens, vineyards, and open habitats that are confined to calcareous substrate. The systematic classification was determined according to morphometric criteria [54]. To provide representative and homogenous data, we designed our experiments to include only newly matured specimens of Roman snail (*Helix pomatia*)*.* The snails were collected, during May 2011, from eight locations in the Banat area, Romania (Timis and Caras-Severin counties), as shown in Figure 6. Being located in a low-anthropized area, away from major sources of pollution [55], the vilage of Salbagelu Nou (Caras-Severin county) has been chosen as reference site (THR). All the other sampling sites shared one set of environmental conditions deemed important for assessing the reliability of Roman snail as bioindicator species in the study unit. Therefore, all locations have been exposed for at least 30 years to industrial pollution, and were located within a 10 km-radius of former and/or actual major sources of contamination (see Table 3). At each plot at least 60 specimens were sampled from a few square meters, and split into three equal samples. To distinguish the newly matured snails from their elder counterparts we used the aperture lip as a benchmark of maturation. Older snails had a hardened, thickened, and turned-out aperture lip*.* In contrast, the newly matured juveniles possess a thinner and softer aperture lip.

**Table 3:** Sampling sites of the environmental matrices (soil, nettle leaves, snail body)

**Sampling site**		**Geographical coordinates**
	**Location/county**	**(latitude/longitude)**
THM1	Timisoara city Landfill	45.6765° lat. N; 21.1626° long. E
	(Parta-Sag/Timis county)	
THM2	South Industrial Platform	45.7112° lat. N; 21.1968° long. E
	(Timisoara/Timis county)	
THM3	North Industrial Platform	45.7420° lat. N; 21.1886° long. E
	(Timisoara/Timis county)	
THM4	East Industrial Platform	45.7763° lat. N; 21.2517° long. E
	(Timisoara/Timis county)	
THM5	Timisoara, Communal Road 64 (DC64)	45.7787° lat. N; 21.2762° long. E
	(Timisoara/Timis county)	
THM6	National Road Resita-Caransebes (DN58)	45.3496° lat. N; 21.9196° long. E
	(Caras-Severin county)	
THM7	Principal Street	45.5053 lat. N; 22.3371 long. E
	(Otelu Rosu/Caras-Severin county)	
THR	Salbagelu Nou	45.5667° lat. N ; 22.0812° long. E
	(Caras-Severin county)	

#### Preparation of snails and conchological measurements

The snails were placed in clean glass containers and fasted for 48 h (the faeces were removed after 24 h). They were sacrificed by freezing (at −20°C) and stored until processing in the lab (chest freezer). After defrosting, the whole soft body was removed from the shell and the viscera and the foot were separated. Only the hepatopancreas (excluding kidney, heart, gonad, and albumen gland) and the foot were studied in this work because these organs are most often reported as the main sites of metal accumulation and storage in snails [20,41]. The samples were analysed in triplicate for each location, and 20 snails were used for each batch. The empty shells were dried with sterile paper towels and kept for further analysis.

Firstly we aimed to find which of the shell main conchological features, shell height (SH) or shell weight (SW), allows a more accurate estimation of shell volume. For each population, ten shells were randomly selected to be measured for determining shell height (SH), shell weight (SW), and shell volume (SV). SH and SW were compiled from the malacological literature [56]; the measurements were performed with a digital caliper (YT 7201, Yato Electronics Co. Ltd, Guangzhou, China) to the nearest 0.01 mm. SV was estimated directly by using a calibrated pipette (vol. = 25 ml, Diffco Din, Witeg, Germany) to fill the shell with distilled deionized water.

Then, we statiscally investigated the presumptive effect of long-term HM exposure on shell height (SH), relative shell height (RSH), and whorl number (WN). The reference population (R snails) was used as a benchmark to reveal intraspecific variation in shell morphology depending on the level of Cu, Zn, Cd, and Pb accumulation in the environmental matrices (soil, nettle leaves, snail foot, snail hepatopancreas). All the shells were measured to determine SH. For each site, the most homogenous 20 shells were selected based on the SH value, and measured to determine SW and WN. These features were also compiled from malacological literature [56]. RSH was calculated as ratio between SH and SW.

All the measurements were performed by the same researcher in the same conditions for all the investigated sites. Every measurement was repeated five times and only the mean value was taken into account.

#### Sampling and preparation of soil and vegetation

At each sampling point numerous Roman snails were observed feeding on nettle (*Urtica dioica*). In addition, irregular holes with smooth edges were often found on nettle leaves in sampling areas revealing *H. pomatia* preference for this perennial plant species; therefore, the selected food chain included nettle as the main food source of Roman snail (*Helix pomatia*). For each location three samples from the top leaves (25 g each) were collected, separated, and rinsed in distilled water to wash off potential air pollutants. The nettle leaves were oven dried at 105°C to constant weight. The dried samples were crushed with a mortar (Isolab SL-1372), passed through a 2 mm sieve, and kept for further analysis in self-sealing sterile paper pouches at room temperature (t = 22°C).

Terrestrial gastropods regularly eat soil [40], and therefore, they accumulate heavy metals not only via food from the food chain, but also via contaminated soil. Because *Helix pomatia* spends its entire life on or in the upper soil horizons, for each location the soil samples were collected (25 g/sample in triplicate) from the top 15 cm layer after removal of vegetation (grass). The fresh soil samples were hand sorted to remove roots and litter and, then dried at room temperature (t = 22°C) for 7 days. Finally, the samples were disaggregated and homogenized before being sieved to 2 mm (soil metal concentration analysis), and then stored at ambient temperature (t = 22°C) for further analysis.

#### Chemical analysis

Chemical analyses were performed separately on each trophic compartment (soil, nettle leaves, snail). The snail meat samples (foot, hepatopancreas) were defrosted (about 5 g/each sample), and oven dried at 105°C for 24 hours. Then, the samples were digested in the Muffle furnace (Nabertherm B150, Lilienthal, Germany); the temperature was stepwise increased up to 550°C till the white ash was formed. The ash was dissolved in 20 mL of 0.5 N HNO_3_ and filtered through ash-free filter paper before analysis. Finally, each sample volume was brought to 50 mL with 30 mL HNO_3_ 0.5 N. The dried nettle samples were similarly processed.

For soil analysis, about 5 g of dried and ground soil per each sample was put in polyethylene tubes (Thermo Scientific Nunc, 30x115 mm, 50 mL). Metals were passed out from soil to solution by wet extraction/proceeding; therefore, each sample was treated with mineral acids (HNO_3_ 0.5 N) at 1:10 soil/nitric acid solution ratio for 24 hours. Then, the samples were centrifugated at 1,500 rpm for 15 min., and for each sample 20 mL of supernatant was transferred in a sterile polyethylene tube (Thermo Scientific Nunc, 30x115 mm, 50 ml) by using a calibrated pipette (vol. = 25 mL, Diffco Din, Witeg, Germany). Finally, each sample volume was bring up to 50 mL with HNO_3_ 0.5 N.

Measurements of HM (Cu, Zn, Cd, Pb) concentrations in environmental matrices (soil, nettle leaves, snail foot, snail hepatopancreas) were carried out in the Environmental Research Test Laboratory, Banat`s University of Agricultural Sciences and Veterinary Medicine from Timisoara, Romania. All samples were weighed on an analytical balance (model TP-214, Denver Instrument Gmbh, Göttingen, Germany) to the nearest 0.1 mg. The digestion solutions (HNO_3_ 0.5 N) were prepared from Merck`Suprapur` nitric acid (65%, ρ = 1.39 g/cm^3^, Merck KGaA, Darmstadt, Germany). The metal concentrations in the filtrate were determined by flame atomic absorption spectrophotometry with high resolution continuum source (Model ContrAA 300, Analytik Jena, Germany), fitted with a specific conditions of particular metal, and they were expressed as miligram per kilogram dry weight (mg kg^-1^ d.w.). Mix standard solutions (1000 mg/L) of Cu, Zn, Cd, and Pb - ICP Multielement Standard solution IV CertiPUR, were purchased from Merck (Merck KGaA, Darmstadt, Germany). Solutions of varying concentrations were prepared for all metals by diluting suitable volumes of standard solutions. Double distilled water (spectroscopic pure) was used for the preparation of reagents and standards. All chemicals were trace metal grade (Suprapur). All glassware was treated with Pierce solution 20% (v/v), rinsed with cold tap water followed by 20% (v/v) nitric acid and then rinsed with double-distilled water. For quality control purposes all blanks and duplicate samples were analyzed during the procedure. NCS Certified Reference Material-DC 85104a and 85105a (China National Analysis Center for Iron&Steel) was analyzed for quality assurance. The percent recovery means were: Cu (102%), Zn (103%), Cd (104%), Pb (96%). The variation coefficients were below 10%. All detection limits (mg kg^-1^) were assessed by using the calibration curve method: Cu (0.12), Zn (0.21), Cd (0.02), Pb (0.04). The blank reagent and standard reference soil were included into each sample batch to verify the accuracy and precision of the digestion procedure and also for subsequent analyses.

#### Statistical analysis

Because of the small size of samples (n = 3), for each environmental matrix (soil, nettle leaves, snail foot and hepatopancreas), a non-parametric test (Kruskal-Wallis one-way ANOVA, df = 7,17) was used for assessing differences in HM accumulation among investigated locations. Similarly, it was determined whether HM accumulation in snail body varied between the snail foot and hepatopancreas (Kruskal-Wallis one-way ANOVA, df = 7,17). To test for significant relationships between HM concentrations within a food chain compartment a bivariate analysis was carried out (Pearson correlation matrix, 16x16 matrix, df = 14). For each environmental matrix (n = 24), HM accumulation was checked to see if it meets the normality assumptions (Shapiro-Wilcoxon test, df = 1,23). The data were then normalized by decimal logarithmation (Shapiro-Wilcoxon test, df = 1,23). Finally, we performed a cluster analysis to classify the investigated sites depending on the level of HM accumulation in snail hepatopancreas. Such statistical analysis produces a 'dendrogram' showing how data points (rows) can be clustered. We used the constrained Ward's method (df = 95) because this alghoritm allows the clusters to be joined such that increase in within-group variance is minimized.

To estimate SV from linear shell dimensions (SH, SW), measured values (n = 80) were normalized by decimal logarithmation (Shapiro-Wilcoxon test, df = 1,79). The relationships existing among them were assessed by a simple correlation analysis (Pearson matrix, df = 8). Next, SH, RSH, and WN values (n = 20) were normalized by decimal logarithmation (Shapiro-Wilcoxon test, df = 1,19). A Brown-Forsythe test (df = 7,153) was conducted to determine if normalized values meet the assumption of homoscedasticity. One Way Analysis of Variance (Anova, df = 7,153) was carried out to test for significant differences in shell size and whorl number among different snail populations. Post hoc analysis used Dunnett’s test (df =1,39). This test is used when several treatment means are each compared to a control/reference mean. Because the sample sizes were different (SH, RSH, WN: n = 20; HM accumulation: n = 3), for each sample only the mean value of each parameter was taken into account to conduct a bivariate analysis (Pearson correlation matrix, 3x16 matrix, df = 14).

Statistical analyses were performed by using Statistica 10 [57] and Past [58] software packages. All data are presented as the mean ± SD. A p value < 0.05 was considered significant.

## Abbreviations

HM: Heavy metals; THR: Reference sampling site; THM1 – THM7: Sampling sites 1-7; SH: Shell height; SW: Shell width; SV: Shell volume; RSH: Relative shell height; WN: Shell whorl number; NC: Normal content; ATV: The alert threshold level.

## Competing interests

The authors declare that they have no competing interests.

## Authors' contributions

DVN, MB, IG, MH, and DMB*, have contributed mainly to the study design, collection of data, sampling of soil, vegetation, and snails, chemical analyses, interpretation of results and preparation of paper. All authors read and approved the final manuscript.
